# Assessment of Tumor Cell Invasion and Radiotherapy Response in Experimental Glioma by Magnetic Resonance Elastography

**DOI:** 10.1002/jmri.29567

**Published:** 2024-08-23

**Authors:** Hannah Fels‐Palesandro, Sophie Heuer, Berin Boztepe, Yannik Streibel, Johannes Ungermann, Chenchen Pan, Jonas G. Scheck, Manuel Fischer, Volker J. Sturm, Daniel D. Azorín, Kianush Karimian‐Jazi, Giacomo Annio, Amir Abdollahi, Ina Weidenfeld, Wolfgang Wick, Varun Venkataramani, Sabine Heiland, Frank Winkler, Martin Bendszus, Ralph Sinkus, Michael O. Breckwoldt, Katharina Schregel

**Affiliations:** ^1^ Department of Neuroradiology Heidelberg University Hospital Heidelberg Germany; ^2^ Clinical Cooperation Unit Translational Radiation Oncology German Cancer Research Center (DKFZ) Heidelberg Germany; ^3^ Department of Neurology and National Center for Tumor Disease (NCT) Heidelberg University Hospital Heidelberg Germany; ^4^ Clinical Cooperation Unit Neurooncology German Cancer Research Center (DKFZ) Heidelberg Germany; ^5^ Clinical Cooperation Unit Neuroimmunology and Brain Tumor Immunology German Cancer Research Center (DKFZ) Heidelberg Germany; ^6^ Department of Biosystems Science and Engineering ETH Zurich Basel Switzerland; ^7^ INSERM UMRS1148 – Laboratory for Vascular Translational Science University Paris Paris France; ^8^ School of Biomedical Engineering and Imaging Sciences King's College London London UK

**Keywords:** brain tumor, glioma invasion, MR elastography, tissue biomechanics, white matter, stiffness

## Abstract

**Background:**

Gliomas are highly invasive brain neoplasms. MRI is the most important tool to diagnose and monitor glioma but has shortcomings. In particular, the assessment of tumor cell invasion is insufficient. This is a clinical dilemma, as recurrence can arise from MRI‐occult glioma cell invasion.

**Hypothesis:**

Tumor cell invasion, tumor growth and radiotherapy alter the brain parenchymal microstructure and thus are assessable by diffusion tensor imaging (DTI) and MR elastography (MRE).

**Study Type:**

Experimental, animal model.

**Animal Model:**

Twenty‐three male NMRI nude mice orthotopically implanted with S24 patient‐derived glioma cells (experimental mice) and 9 NMRI nude mice stereotactically injected with 1 μL PBS (sham‐injected mice).

**Field Strength/Sequence:**

2D and 3D T2‐weighted rapid acquisition with refocused echoes (RARE), 2D echo planar imaging (EPI) DTI, 2D multi‐slice multi‐echo (MSME) T2 relaxometry, 3D MSME MRE at 900 Hz acquired at 9.4 T (675 mT/m gradient strength).

**Assessment:**

Longitudinal 4‐weekly imaging was performed for up to 4 months. Tumor volume was assessed in experimental mice (n = 10 treatment‐control, n = 13 radiotherapy). The radiotherapy subgroup and 5 sham‐injected mice underwent irradiation (3 × 6 Gy) 9 weeks post‐implantation/sham injection. MRI‐/MRE‐parameters were assessed in the corpus callosum and tumor core/injection tract. Imaging data were correlated to light sheet microscopy (LSM) and histology.

**Statistical Tests:**

Paired and unpaired *t*‐tests, a *P*‐value ≤0.05 was considered significant.

**Results:**

From week 4 to 8, a significant callosal stiffening (4.44 ± 0.22 vs. 5.31 ± 0.29 kPa) was detected correlating with LSM‐proven tumor cell invasion. This was occult to all other imaging metrics. Histologically proven tissue destruction in the tumor core led to an increased T2 relaxation time (41.65 ± 0.34 vs. 44.83 ± 0.66 msec) and ADC (610.2 ± 12.27 vs. 711.2 ± 13.42 × 10^−6^ mm^2^/s) and a softening (5.51 ± 0.30 vs. 4.24 ± 0.29 kPa) from week 8 to 12. Radiotherapy slowed tumor progression.

**Data Conclusion:**

MRE is promising for the assessment of key glioma characteristics.

**Evidence Level:**

NA

**Technical Efficacy:**

Stage 2

Glioblastoma is the most common primary malignant brain tumor and is characterized by a particularly invasive growth.^1^ MRI is the key element for noninvasive diagnosis and therapy monitoring.^2^ Yet, clinically established MRI sequences do not allow for a precise tumor delineation, especially regarding its invasion zones.^3^ This is a diagnostic dilemma, as areas with microscopic, non‐MRI detectable tumor cell infiltration cannot be adequately treated and can be the starting point for recurrence.^4^

The S24 patient‐derived xenograft (PDX) model has been extensively characterized with an emphasis on its invasive growth pattern and the formation of a multicellular tumor‐to‐tumor and neuron‐to‐tumor network mediated by neurite‐like membrane protrusions called tumor microtubes (TM).^5^, ^6^ The progressive development of TMs leads to structurally and functionally highly interconnected tumor cell networks where the TM‐connected tumor cells are particularly therapy‐resistant, while unconnected tumor cells are more susceptible to antitumoral treatments such as irradiation and chemotherapy.^5^ On MRI, S24 glioma show subtle T2w hyperintensity and a progressive mass effect. Tumors present with only little to none contrast‐enhancement at late disease stages and show only rarely microhemorrhages or neo‐angiogenesis.^7^ In contrast to other models such as syngeneic Gl261 mouse glioma or U87MG PDX glioma, the extent of S24 glioma cannot reliably be detected by MRI,^8^ mimicking the clinical diagnostic dilemma and rendering the S24 model particularly suited to study tumor cell invasion.

As invading tumor cells and glioma growth alter the brain's parenchymal organization, we hypothesized that imaging techniques sensitive to tissue microstructure could detect tumor cell invasion. To test this hypothesis, we evaluated parameters derived from diffusion tensor imaging (DTI) and MR elastography (MRE). Tumor volume and T2 relaxation time as a surrogate for tissue water content were analyzed as well. DTI, which assesses the diffusion of water molecules within a given tissue^9^ thereby indirectly reflecting tissue microstructure, allows for the calculation of the apparent diffusion coefficient (ADC) and fractional anisotropy (FA). ADC has been suggested as a proxy of glioma cell density,^3^ while FA has been proposed as a marker for tumor cell invasion.^10^ MRE enables the quantification of biomechanical tissue properties in vivo^11^, ^12^ and thereby renders information on tissue microstructure complimentary to DTI. Here, the magnitude of the complex shear modulus IG*I, in the following referred to as “stiffness,” and the phase angle Y, informing about the relative contributions of elastic and viscous components to |G*|,^13^ were assessed. MRE has been clinically established for liver imaging^14^ and has recently shown potential in neuro‐oncology.^15^, ^16^, ^17^

The aim of this study was to assess the capability of MRI‐ and MRE‐metrics to detect tumor cell invasion in the corpus callosum (CC), a structure shown to be an invasion route for S24 glioma cells at early time points^18^ and which is also affected in most patients with glioblastoma.^19^ Further aims were to quantify these parameters in the tumor core, to investigate the effects of radiotherapy on imaging metrics, and to validate the findings by 3D light sheet microscopy (LSM) and histology.

## Materials and Methods

### Glioma Model

The patient‐derived glioblastoma cell line S24 (GBMSC; IDH‐wildtype as evidenced by 850k methylation array and scRNA‐sequencing) was kept under stem‐like, serum‐free conditions in spheroid cell culture as previously described.^20^, ^21^ The cells were cultured in DMEM‐F12 medium (Invitrogen, 11330‐032) that was supplemented with 20 μL/mL B27 supplement (Invitrogen, 12587‐010), 5 μg/mL insulin (Sigma‐Aldrich, I9278), 5 μg/mL heparin (Sigma‐Aldrich, H4784), 20 ng/mL epidermal growth factor (rhEGF; R&D Systems, 236‐EG), and 20 ng/mL basic fibroblast growth factor (bFGF; Thermo Fisher Scientific, PHG0021).^20^, ^21^ Stable transduction with the lentiviral vector pLKO.1‐puro‐CMV‐TurboGFP_shnon‐target‐vector (custom made, Sigma‐Aldrich) induced green fluorescent protein (turbo‐GFP) expression in the GBMSCs by incubation with 10 μg/mL polybrene (TR‐1003‐G, Merck Millipore) and the lentiviral particles for 24 hours.^22^

Specific and Opportunistic Pathogen Free (SOPF) male NMRI mice were bought from Charles River Laboratories (Sulzfeld, Germany; minimum age 6 weeks) and used for experiments 1 week later. Mice were housed in the specific pathogen‐free (SPF) animal facility of the DKFZ Heidelberg with 12‐hour/12‐hour‐light/dark‐cycle, and food and water *ad libitum*. One‐hundred‐thousand S24 cells in 1 μL phosphate buffered saline (PBS, Sigma‐Aldrich) or only 1 μL of PBS (sham injection) were stereotactically implanted in the right striatum. Mice were anesthetized with ketamine/xylazine (kx) during the procedure. Implantation was performed using a Hamilton syringe with a fine step motor.^23^ All animal protocols were performed in compliance with the laboratory animal research guidelines and were approved by the governmental authorities (animal protocol: 35‐9185.81/G‐111/21, Regierungspräsidium Karlsruhe, Germany).

### Study Design

Experimental mice were stratified into a treatment‐control group (N = 10) or a radiotherapy group (N = 13) based on T2w tumor burden in week 8 assuring matching tumor volumes between the groups before treatment (tumor volume quantified after manual segmentation on 2D T2w; consensus reading of three neuroradiologists: H.F.‐P., K.S. and M.O.B.). The radiotherapy group received 6 Gy tumor irradiation on three consecutive days (cumulative dose 18 Gy) in week 9 after tumor cell implantation as previously described.^5^ Details are provided in the Supporting Information.

MRI and MRE were performed in weeks 4, 8, and 12 and for a subgroup of irradiated animals (N = 5) in week 16 post‐implantation. After the last MRI examination, mice were anesthetized with a lethal dose of kx and transcardially perfused with PBS followed by 4% paraformaldehyde (PFA). Brains were harvested and prepared for whole brain tissue clearing and subsequent LSM as previously described.^7^, ^24^ After completion of LSM, rehydration and histology were performed (N = 7). All presented data is pooled from two individual experimental series, the first series with 8 tumor‐bearing mice (treatment‐control group N = 5, radiotherapy group N = 3), second series with 15 mice (treatment‐control group N = 5, radiotherapy group N = 10).

**FIGURE 1:**
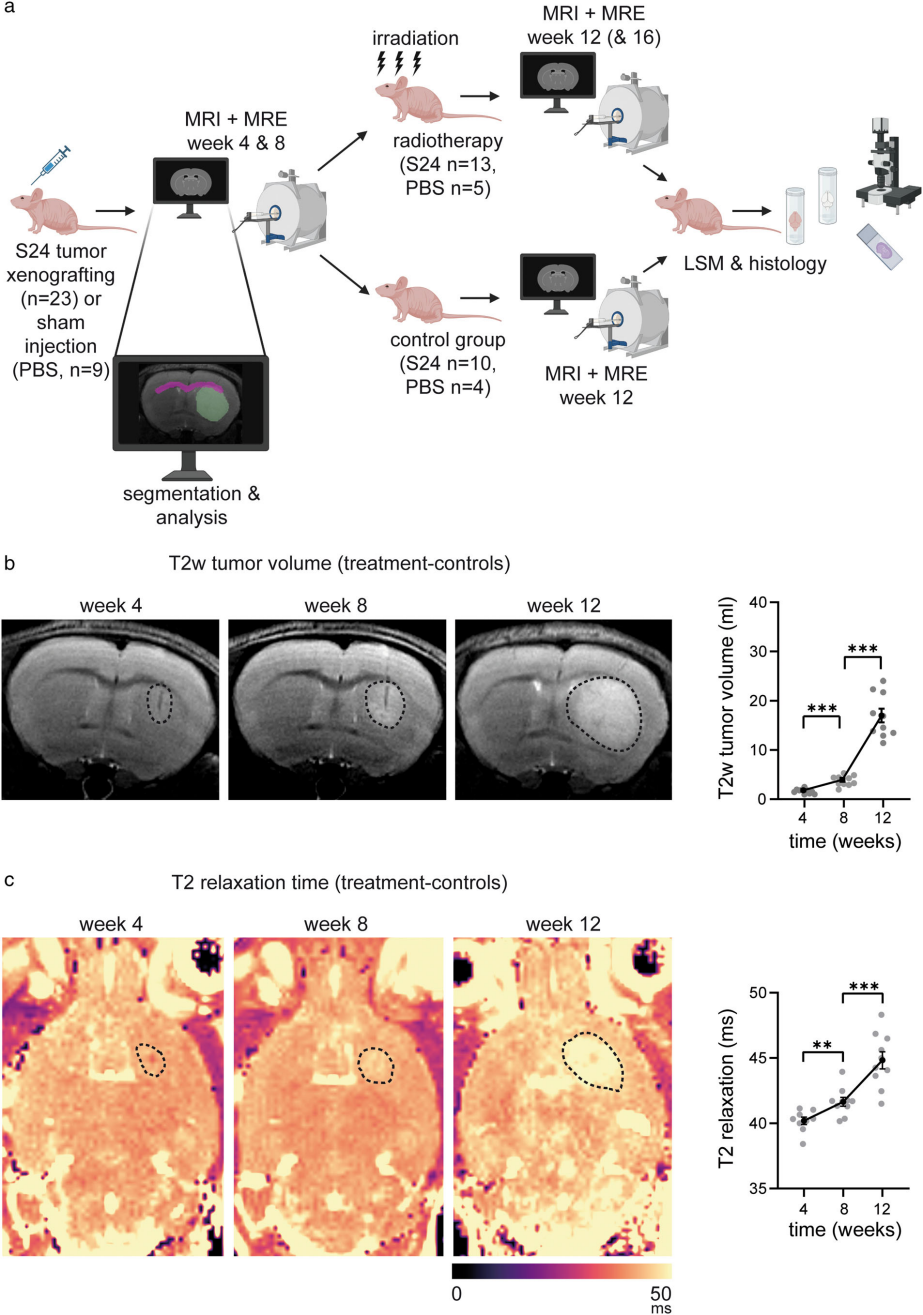
Study design and tumor core growth on structural MRI. (**a**) Illustration of the study design and examples of segmented regions placed in the tumor core and corpus callosum. Imaging was performed every 4 weeks starting in week 4 after S24 PDX implantation (N = 23 mice) or PBS sham injection (N = 9 mice). Animals were distributed to the photon radiation or treatment‐control group following the baseline scan in week 8 and monitored until week 12 (treatment‐control group, N = 10, irradiation group, N = 13) or 16 (irradiation group, N = 5). (**b**) Representative illustration and quantification of tumor growth in the treatment‐control group on longitudinal T2w imaging in weeks 4, 8, and 12. The T2w hyperintense tumor is highlighted by the dotted lines. (**c**) Longitudinal illustration and quantification of T2 relaxometry maps in treatment‐controls from week 4 to week 12 (dotted lines indicate the tumor core). Statistical testing was performed by paired *t*‐tests comparing the different time points, **P ≤ 0.01; ***P ≤ 0.001. N = 10 mice (treatment‐controls). Illustration in (a) was created with Biorender (www.biorender.com).

Sham control animals received a 1 μL PBS injection into the right striatum (N = 9) to control for possible biomechanical alterations induced by the injection tract. These mice underwent MRI and MRE in weeks 4, 8, and 12 post‐injection. Five of these animals received irradiation of the injection site (3 × 6 Gy; cumulative dose 18 Gy) in week 9 post‐injection. The study design is shown in Fig. 1a.

### MR Imaging

MRI and MRE were performed on a 9.4 T Bruker small animal MRI scanner (BioSpec 94/20 USR, Bruker BioSpin GmbH, Ettlingen, Germany; gradient strength 675 mT/m) with an 8.4 cm body coil for transmission and a 2 × 2 surface array coil for reception. Animals were anesthetized with 1.5%–2.5% isoflurane (Baxter, Unterschleißheim, Germany) in 100% O_2_ and anesthesia was maintained with 1.0%–1.5% isoflurane in 100% O_2_ for the duration of the scan. For multiparametric MRI, mice were placed prone on a standard Bruker MRI bed and for MRE on a custom‐built bed, both equipped with a water heating system at 39°C to maintain the animal's body temperature. The MRE bed was a modification of previously published hardware versions.^25^, ^26^, ^27^ Briefly, a flexible nylon rod connected an external electromagnetic shaker (Mini‐shaker 4810, Brüel & Kjaer, Naerum, Denmark) with a head cradle fixating the animal's head. Vibrations at 900 Hz frequency were generated by the shaker and transmitted via the rod to induce mechanical shear waves in the mouse brain. Synchronization of vibrations and MRE acquisition was obtained by detecting a TTL trigger pulse from the MRI scanner with a function generator that controlled the shaker. Throughout the scan, the respiration rate was monitored with a breathing surface pad controlled by an in‐house developed LabVIEW program (National Instruments Corporation). After standard shimming and acquisition of a B0 field map, a 2D T2w Rapid Imaging with Refocused Echoes (RARE), a 3D T2w RARE with an isotropic resolution of 120 μm, an echo planar imaging (EPI) DTI sequence with a *b*‐value of 1500 s/mm^2^, 30 directions, a gradient duration of 3 msec and a gradient separation of 9 msec with max. 464 mT/m diffusion gradient strength were acquired. 3D MRE based on a spin‐echo multi‐slice multi‐echo (MSME) sequence at a vibration frequency of 900 Hz was performed with 18 motion‐encoding gradient cycles. In all tumor‐bearing animals, a MSME T2 relaxometry with 30 effective echo times was additionally acquired. Further parameters of the MRE and MRI sequences are provided in Table 1.

**TABLE 1:** Parameters of MRI and MRE Sequences

Sequence	TR (msec)	TE (msec)	Flip Angle	Averages	Acquisition Matrix	FOV (mm)	Slice Thickness (mm)	Number of Slices	Duration
3D T2w TurboRARE	1800	72.5	90	1	200 × 200	20 × 10 × 12	0.1	120	10 min 48 s
2D T2w TurboRARE; axial	2500	33	90	2	256 × 256	20 × 20	0.7	20	2 min 40 s
2D EPI DTI; axial	3400	20	90	1	96 × 128	12 × 15	0.7	17	7 min 56 s
2D MSME T2 relaxometry; axial	3500	7, 14, 21, 28, 35, 42, 49, 56, 63, 70, 77, 84, 91, 98, 105, 112, 119, 126, 133, 140, 147, 154, 161, 168, 175, 182, 189, 196, 203, 210	90	1	100 × 75	20 × 15	0.5	14	4 min 22 s
2D T2w TurboRARE; axial (for MRE‐planning)	1250	33	90	2	128 × 128	19.2 × 19.2	0.3	9	5 min 20 s
3D MSME MRE; 900 Hz vibration frequency; axial	1500	26.67	90	1	64 × 64	19.2 × 19.2	0.3	9	19 min 12 s

DTI = diffusion tensor imaging; EPI = echo planar imaging; FOV = field of view; MRE = magnetic resonance elastography; MSME = multi‐slice multi‐echo; RARE = rapid acquisition with relaxation enhancement; TE = echo time; TR = repetition time.

### Tissue Clearing and LSM

After the last imaging time point, animals received a lethal intraperitoneal injection of 400 μL kx and were intracardially perfused with 30 mL PBS followed by 30 mL PFA. Brains were harvested and immersed in PFA at 4°C overnight followed by storage in PBS. Brains were optically cleared based on the iDISCO protocol^28^ with previously established adaptions by our lab.^24^ Samples were treated with a primary mouse anti‐mouse TurboGFP antibody (#TA150041, ThermoFisher) followed by a goat anti‐mouse IgG (H + L) secondary antibody (Alexa Fluor 568, #A‐11004, ThermoFisher).

Optically cleared brains were scanned with a light sheet microscope (LCS SPIM, Luxendo‐Bruker or UltraMicroscope II, Miltenyi Biotec; Heidelberg, Germany) using respectively a 2.0× or 1.0× objective lens and combined lasers (excitation wavelength at 488, 561, and 642 for LCS SPIM and 488, 568, and 647 for Ultramicroscope II with their respective filters). A pixel size of 6 × 6 μm was used for image acquisition and tiling z‐stack scans with 10 μm (LCS SPIM) or 5 μm (Ultramicroscope II) step size were performed to cover the entire brain sample. Images were exported as series of tagged image files (TIF) and then downsampled using the bin transform of open‐source FIJI software (FIJI/ImageJ for Windows, version 2.0, www.imagej.net ^29^) with a shrink factor of 2 × 2 × 2 averaging voxels within the 2 × 2 × 2 region into one voxel resulting in a downsampled resolution of 12 × 12 × 20 μm (LCS SPIM) or 12 × 12 × 10 μm (Ultramicroscope II).

### Rehydration and Histology

Following LSM a subset of brains (N = 7, three treatment‐controls and four irradiated animals) underwent rehydration, cryo‐sectioning at a slice thickness of 10 μm and standard hematoxylin and eosin (H&E) and Alcian blue staining. Details are provided in the Supporting Information. Imaging was performed on a Zeiss Axio Scan.Z1 with a 20× magnification (Zeiss, Germany).

Immunofluorescence (IF) was performed on 400 μm brain tissue sections from untreated tumor‐bearing mice sacrificed in week 11 (N = 3, tissue samples provided by V.V.). The GFP signal was enhanced by counterstaining against GFP. Details can be found in the Supporting Information. Confocal images were recorded as z‐stacks with a Nikon A1R Laser Scanning Microscope (Rights Reserved to Nikon Imaging Center Heidelberg), at 4× and 20× magnification for DAPI and Alexa 488.

### Data Reconstruction and Image Analysis

MRE data were reconstructed using dedicated in‐house software (ROOT environment, CERN; Meyrin, Switzerland) according to published algorithms.^12^, ^30^ Succinctly, per slice unwrapping of phase data and then alignment between slices was performed. As four wave phases were acquired, a 4‐point Fourier transform was calculated and the first harmonic amplitude was employed to represent the displacement amplitude. The curl operator was applied on the displacement data to remove the compressional component of the waves. Calculation of spatial derivatives was performed and used to inverse the Helmholtz equation. MRE‐parameters were displayed in color‐coded maps (elastograms) and then exported in NIfTI format.

IG*I, the magnitude of the complex shear modulus, is composed of an elastic (shear modulus, *G*_*d*_) and a viscous component (loss modulus, *G*_*l*_) and was calculated as $\left|G*\right|=\sqrt{{G_{d}}^{2}+{G_{l}}^{2}}$. The relative contributions of elasticity and viscosity to the shear modulus are expressed in the phase angle by $Y=\frac{2}{\pi }\;\text{atan}\left(\frac{G_{l}}{G_{d}}\right)$. At the extremes, a given material is purely elastic (*Y* = 0) or purely viscous (*Y* = 1).

ADC and FA maps were calculated from DTI raw data and exported in DICOM‐format using a customized script (MATLAB R2020a, 64‐bit version for Windows, The MathWorks Inc., Natick, MA, USA). Maps of T2 relaxation time were obtained using a customized MATLAB script, which included noise filtering with removal of all voxels exceeding four SDs of noise level of each dataset before fitting the data.

Processing and correlative analysis of MRI, MRE, and LSM data were performed with open‐source 3D Slicer (3D Slicer for Windows and macOS, versions 5.1.0 and 5.4.0, www.slicer.org ^31^) in either NIfTI or Nrrd format. First, qualitative assessment of MR images including evaluation of tumor related mass effect, homogeneity of T2w signal or tumor delineation on elastograms was performed by consensus reading of three neuroradiologists (H.F.‐P., K.S., and M.O.B., with 4 to 12 years of experience in interpreting clinical and preclinical MRI scans). This was followed by a qualitative evaluation of S24 glioma cell distribution on LSM‐data, again by consensus reading of H.F.‐P., K.S., and M.O.B. For quantitative analyses, maps of ADC, FA and T2 relaxation time, elastograms and 2D T2w images were co‐registered to the respective 3D T2w images of each mouse per time point with a linear rigid transform. Downsampled LSM‐data of each mouse were co‐registered to the respective 3D T2w acquired in either week 12 or for a subset of mice in week 16 using a warping transform generated with a manual anatomical‐landmark based registration approach.^8^

Tumor core volumes were quantified on 2D T2w images for each time point by manual segmentation of hyperintense areas by consensus reading (H.F.‐P., K.S. and M.O.B.). To further assess infiltration zones, the corpus callosum was manually segmented for each time point on T2w images (consensus reading of H.F.‐P., K.S. and M.O.B.). Mean values of ADC, FA, |G*|, Y, and T2 relaxation time were determined within these regions (tumor core, corpus callosum). In sham‐injected mice, the CC, the needle tract and a similar sized area in the contralateral hemisphere were segmented analogously and mean values of ADC, FA, |G*|, and Y were obtained for each region.

### Histology Analysis

H&E staining of tumor‐bearing mice were first evaluated qualitatively (consensus reading of H.F.‐P., J.U., and M.O.B. with 4 to 15 years of experience in analyzing histology slides) with FIJI and then quantified with open‐source QuPath software (QuPath for Windows, version 0.5.1, https://qupath.github.io ^32^). Briefly, two equally sized ROIs were placed in the tumor core and analogously in the contralateral hemisphere. The absolute cell number per ROI was then quantified with the “Positive Cell Detection” plugin. In a last step, the mean cell density per 100 μm^2^ was calculated for both ROIs.

Brain slices stained with Alcian blue were quantified in FIJI using a sample guided color‐thresholding pipeline. First, images (czi‐format) were transformed to fit the RGB color space and two equally sized ROIs were placed in the tumor core and in the contralateral hemisphere. Then, 20 RGB pixel values that conformed with the Alcian blue hue were obtained from the tumor‐containing region and a threshold range was calculated as the mean ± 2 SD for each color‐channel. The calculated threshold was then applied to both ROIs and a readout was generated by FIJI's “Analyze Particles” function. Results were read as the area within threshold range per ROI in % and validated by consensus reading between J.U., M.O.B., and H.F.‐P.

For IF staining, maximal projection images were generated from Z‐Stack files with FIJI and adjusted for brightness and contrast. Slides were then analyzed by consensus reading of H.F.‐P., J.U., and M.O.B.

### Statistical Analysis

GraphPad Prism (version 10.1.1 for macOS, GraphPad Software, La Jolla, CA, USA) was used for statistical analysis. Given the study's exploratory nature and consequently its inherent problem of adequate multiple test adjustment,^33^ paired *t*‐tests comparing the different time points were conducted to evaluate the temporal evolution of tumor volume, T2 relaxation time, FA, ADC, |G*|, and Y in each subgroup (tumor bearing groups, sham‐injected group), unpaired *t*‐tests were employed to compare the difference (Δ) between two successive time points or the absolute values at a given point between the different groups. Data are represented as individual values (dots or rectangles in gray or black) and as their respective mean ± standard error of mean (SEM). A *P*‐value ≤0.05 was considered significant in the realm of our exploratory study.

## Results

### Tumor Volumetry Based on T2w Images and Evaluation of T2 Relaxation Times

In the 23 experimental mice, glioma growth was appreciated as faint T2w‐hyperintensity around the injection tract 4 weeks after implantation (Fig. 1b). Over time, T2w‐hyperintensity gradually increased, correlating with a significant increase in T2 relaxation time (week 4: 40.19 ± 0.28 msec, week 8: 41.65 ± 0.34 msec, week 12: 44.83 ± 0.66 msec; Fig. 1c). Overall, the tumor core appeared rather uniform on T2w images and T2 relaxometry (Fig. 1b,c). Tumor subregions or infiltration zones were not delineable. While the tumor core remained small at early time points with a mean volume of 1.6 ± 0.15 mm^3^ in week 4 and of 3.76 ± 0.33 mm^3^ in week 8 after xenografting, it increased exponentially and significantly from week 8 to week 12 (16.88 ± 1.40 mm^3^; Fig. 1b). In week 12, the tumor core induced a moderate mass effect compressing adjacent brain structures such as the corpus callosum or the ventricular system (Fig. 1b).

### Assessment of MRI‐ and MRE‐Metrics in the Corpus Callosum

**FIGURE 2:**
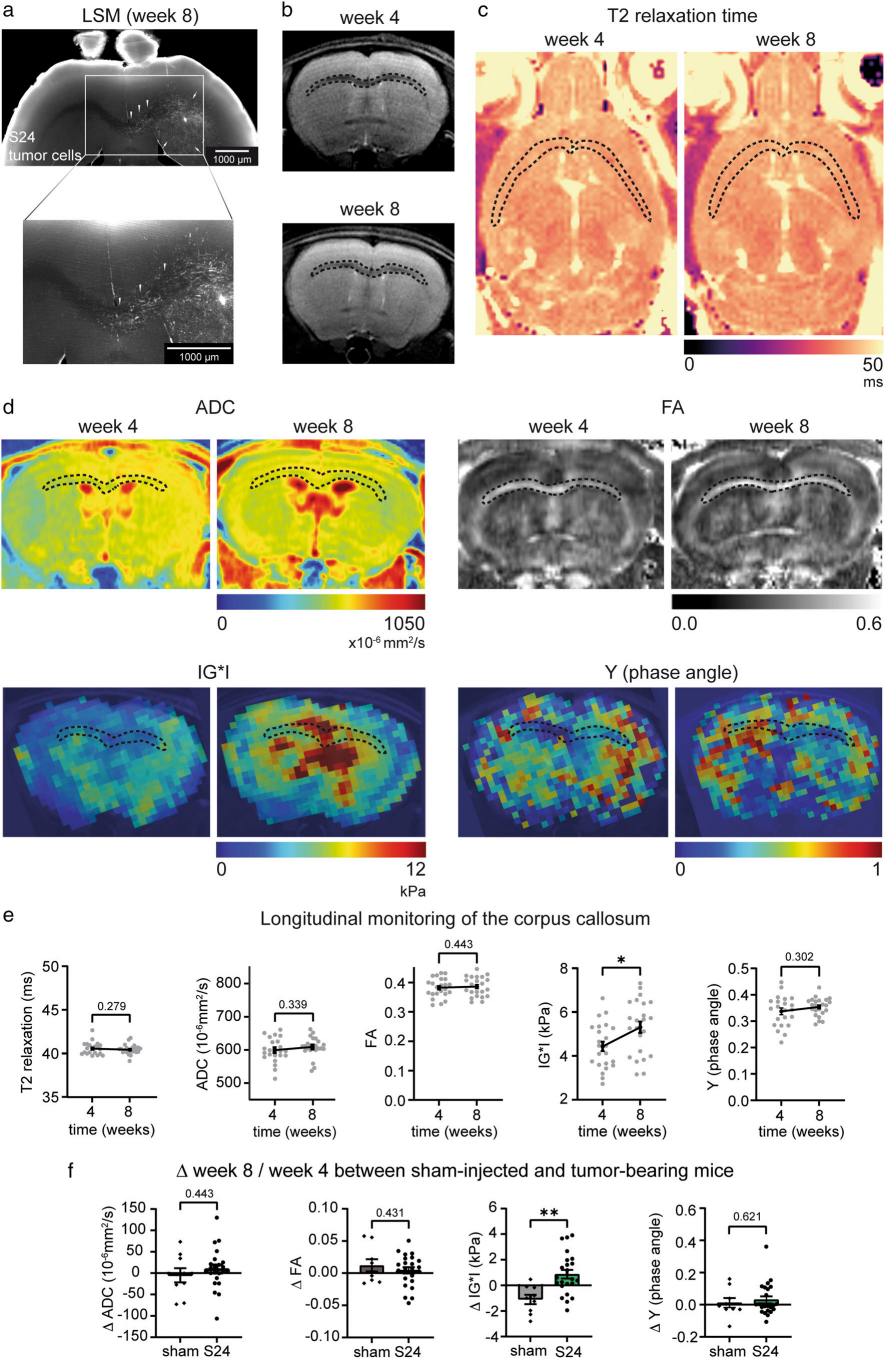
Analysis of T2 relaxation time, DTI‐ and MRE‐metrics in the corpus callosum. (**a**) A representative light sheet image after tissue clearing shows a dense tumor cell network in the tumor core (arrows) with infiltration of the corpus callosum (arrowheads) in week 8. (**b**, **c**) No callosal signal alterations are observed on T2w and T2 relaxation maps at the same timepoint (dotted lines indicate the CC). (**d**) Visualization of ADC and FA maps and elastograms of IG*I and Y (dotted lines highlight the CC). (**e**) Quantification of callosal T2 relaxation time, ADC, FA, IG*I, and Y from week 4 to week 8 in tumor‐bearing mice. (**f**) Evolution of ADC, FA, IG*I, and Y from week 4 to week 8 (Δ week 8/week 4) compared to PBS sham‐injected mice. Statistical analysis was performed with paired *t*‐tests when comparing different time points within a group and unpaired *t*‐tests when comparing different groups, **P ≤ 0.01. N = 23 (tumor‐bearing mice), N = 9 (PBS sham‐injected mice).

Contralateral hemisphere infiltration via the CC was detectable by LSM in week 8 post‐injection (Fig. 2a). Yet, this invasion was not observable on T2w images (Fig. 2b). Also, mean callosal T2 relaxation time, ADC and FA did not show alterations in week 8 after tumor implantation (T2 relaxation time in week 4: 40.5 ± 0.16 msec vs. week 8: 40.44 ± 0.12 msec, *P* = 0.279; ADC in week 4: 598.9 ± 7.57 × 10^−6^ mm^2^/s vs. week 8: 608.5 ± 6.27 × 10^−6^ mm^2^/s, *P* = 0.339; FA in week 4: 0.382 ± 0.007 vs. week 8: 0.386 ± 0.007, *P* = 0.443; Fig. 2c–e). In contrast, MRE revealed a significant increase of CC stiffness (mean IG*I in week 4: 4.44 ± 0.22 kPa vs. week 8: 5.31 ± 0.29 kPa; Fig. 2d,e). The callosal phase angle Y was stable over time (week 4: 0.337 ± 0.012, week 8: 0.354 ± 0.007, *P* = 0.302; Fig. 2d,e).

When comparing the evolution of callosal stiffness from week 4 to week 8 between tumor‐bearing and sham‐injected control mice, there was a significant stiffening of the CC in tumor‐bearing mice, which was not present in sham‐injected controls (Δ week 8/week 4 in tumor‐bearing animals: 0.88 ± 0.34 kPa vs. in sham‐injected mice: −1.1 ± 0.36 kPa; Fig. 2f). There were no differences in mean callosal ADC or FA when comparing tumor‐bearing and sham‐injected mice (ADC: Δ week 8/week 4 in tumor‐bearing animals: 9.59 ± 9.81 × 10^−6^ mm^2^/s vs. in sham‐injected mice: −5.01 ± 16.58 × 10^−6^ mm^2^/s, *P* = 0.443; FA: Δ week 8/week 4 in tumor‐bearing animals: 0.0041 ± 0.0052 vs. in sham‐injected mice: 0.0122 ± 0.0094, *P* = 0.431; Fig. 2f).

**FIGURE 3:**
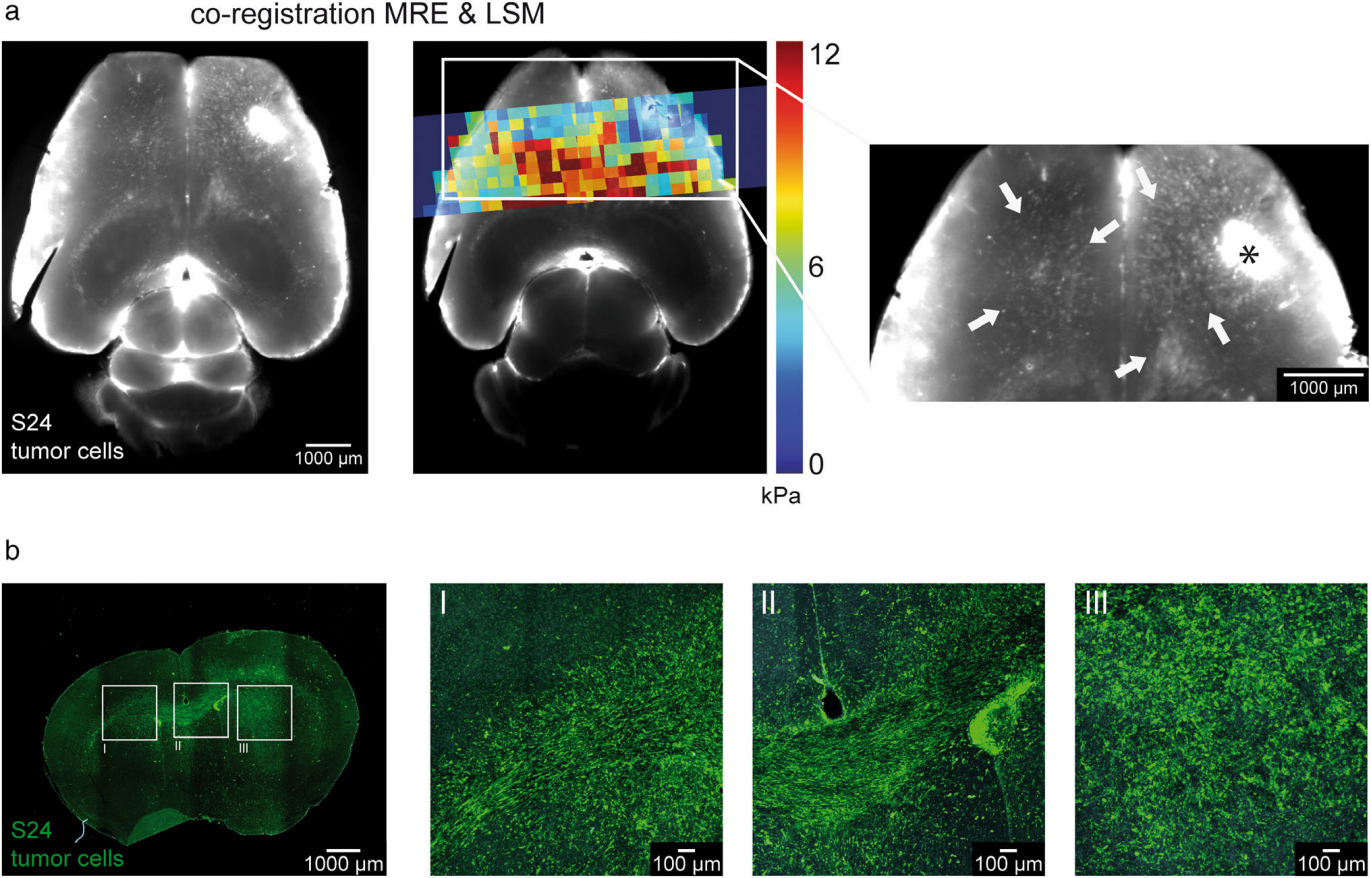
Tumor cell invasion on LSM and Immunofluorescence. (**a**) Visual association between tumor cell invasion and areas of increased tissue stiffness at later time points (week 12 and 16) in the ipsi‐ and contralateral hemisphere (areas of pronounced tumor cell invasion are indicated by the arrows, the asterisk marks the injection tract). (**b**) Representative confocal micrograph of immunofluorescence staining of S24 tumor cells from week 11 with magnified images of the contralateral (I) and ipsilateral (II) invaded CC and the tumor core (III).

Co‐registered MRE and LSM datasets showed a visual association between tumor cell infiltration and increased tissue stiffness of the infiltrated gray and white matter of the ipsi‐ and contralateral hemisphere at later time points (week 12 and 16; Fig. 3a). In line with these findings, IF staining from week 11 showed differences in architecture and organization of tumor cells between the invasion sites and the tumor core. While invading tumor cells showed more TMs and were in vast majority orientated along fiber tracts, the tumor core displayed a higher cell density with a more disorganized architecture (Fig. 3b).

### Assessment of MRI‐ and MRE‐Parameters in the Tumor Core

**FIGURE 4:**
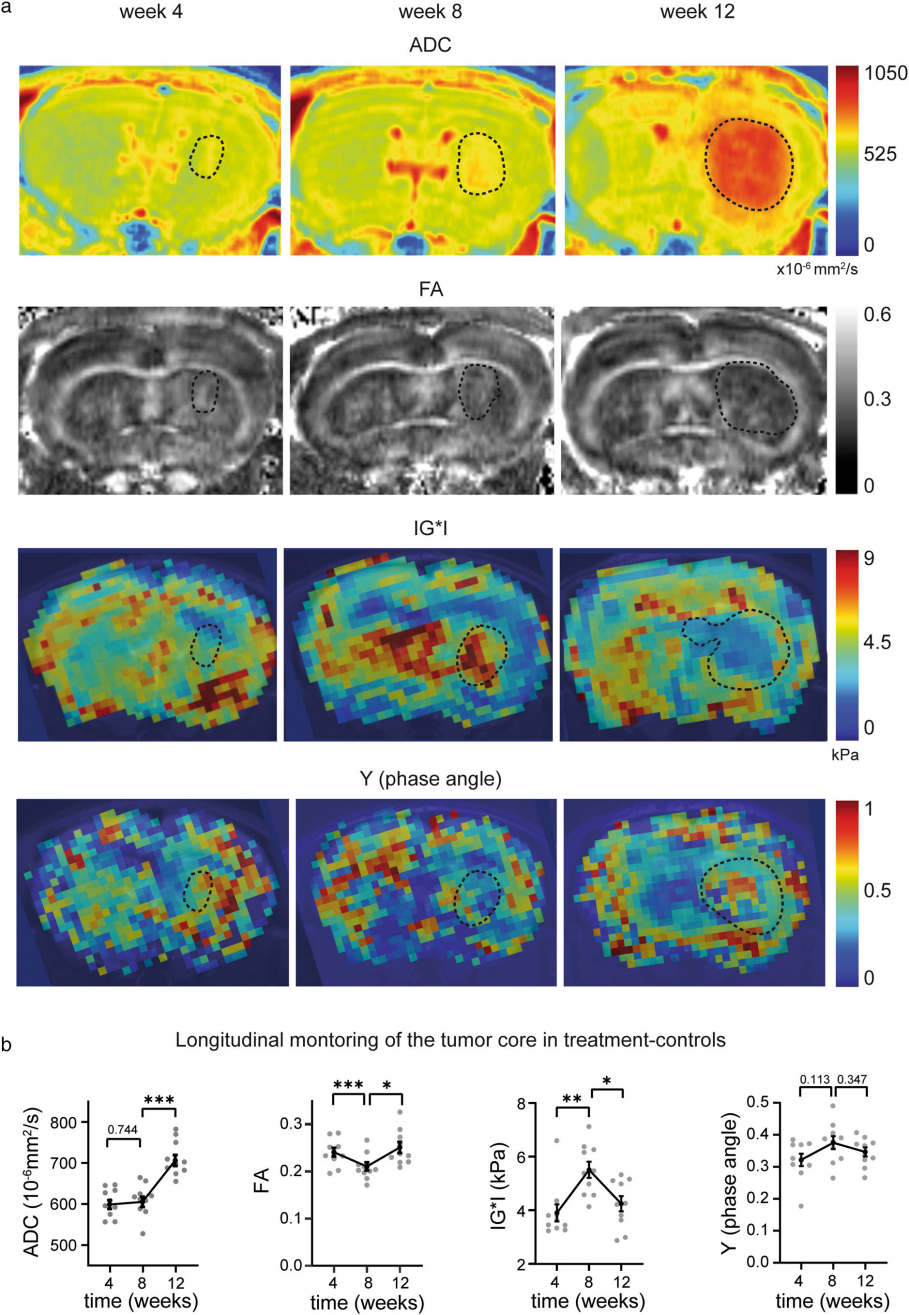
DTI‐ and MRE‐dynamics in the T2w hyperintense tumor core of tumor‐bearing treatment‐controls. (**a**) Representative ADC and FA maps and elastograms of IG*I and Y from week 4 to 12, dotted lines indicate the tumor core. (**b**) Longitudinal quantification of ADC, FA, IG*I, and Y in the tumor core. Statistical analysis was performed with paired *t*‐tests comparing the different time points, **P ≤ 0.01; ***P ≤ 0.001. N = 10 mice (treatment‐controls).

The ADC increased significantly within the tumor core at late time points (ADC week 8: 610.2 ± 12.27 × 10^−6^ mm^2^/s vs. week 12: 711.2 ± 13.42 × 10^−6^ mm^2^/s; Fig. 4a,b).

The FA in the tumor core first decreased and then increased from week 8 to 12 (week 4: 0.2398, week 8: 0.2097, week 12: 0.2479; Fig. 4a,b).

The evolution of tumor core stiffness was biphasic. There was a significant stiffness increase in the tumor core from week 4 to 8, (IG*I week 4: 3.9 ± 0.32 kPa, week 8: 5.51 ± 0.30 kPa; Fig. 4a,b). This increase was followed by a progressive, significant softening of the tumor core in week 12 (IG*I = 4.24 ± 0.29 kPa) with heterogeneous, stiffer clusters remaining in the tumor periphery (Fig. 4a,b). The tumor core phase angle remained stable over time (week 4: 0.322 ± 0.019, week 8: 0.375 ± 0.020, week 12: 0.346 ± 0.014, week 4 vs. 8, *P* = 0.113, week 8 vs. 12, *P* = 0.347; Fig. 4a,b).

**FIGURE 5:**
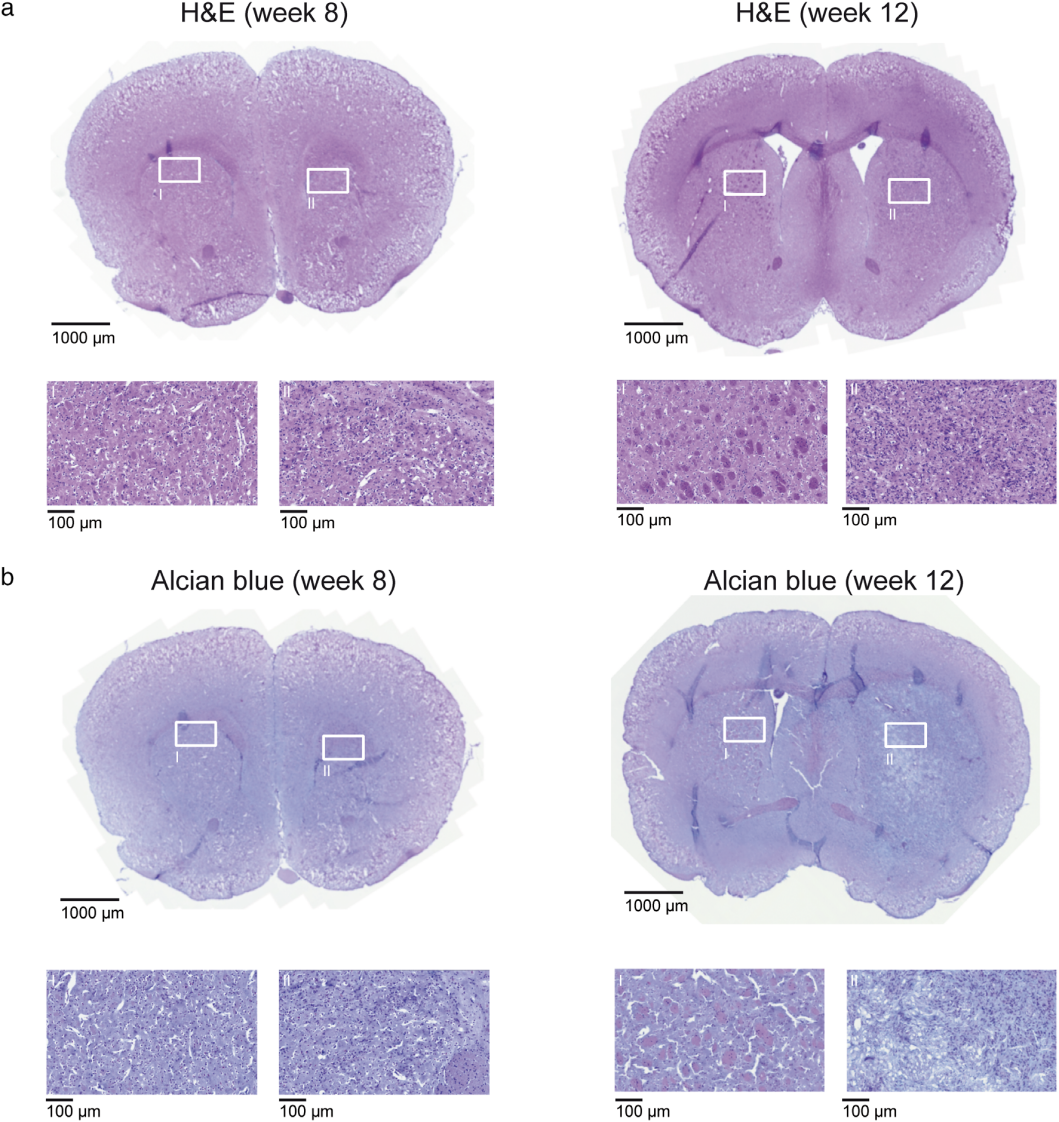
Histology of tumor‐bearing treatment‐controls from week 8 and week 12. (**a**) Hematoxylin & Eosin (H&E) staining of treatment‐controls from week 8 and 12 with magnified illustrations of the tumor core and the contralateral hemisphere. (**b**) Alcian blue staining of treatment‐controls from week 8 and week 12 with magnified illustrations of the tumor core and the contralateral hemisphere.

Histologically, a marked increase in cell density was observable within the tumor core when comparing week 8 to 12 (47.2 cells/100 μm^2^ vs. 59.9 cells/100 μm^2^ on representative slices for each time point; Fig. 5a). In week 8, S24 cells were interspersed with relatively preserved brain tissue, eg, white matter tracts were delineable within the tumor core. In week 12, the normal architecture of brain tissue was no longer identifiable (Fig. 5a). Furthermore, Alcian blue staining revealed a pronounced destruction of the ECM with increased amounts of stained glycosaminoglycans and mucopolysaccharides (average percentage of disrupted ECM in the tumor core 0.40% in week 8 vs. 11.27% in week 12; Fig. 5b).

Sham‐injected mice showed no significant differences in ADC, FA, IG*I or Y between the injection tract and an equally sized area in the contralateral hemisphere at each measurement point (Fig. S1 in the Supplemental Material).

### Effects of Radiotherapy on Imaging Parameters

**FIGURE 6:**
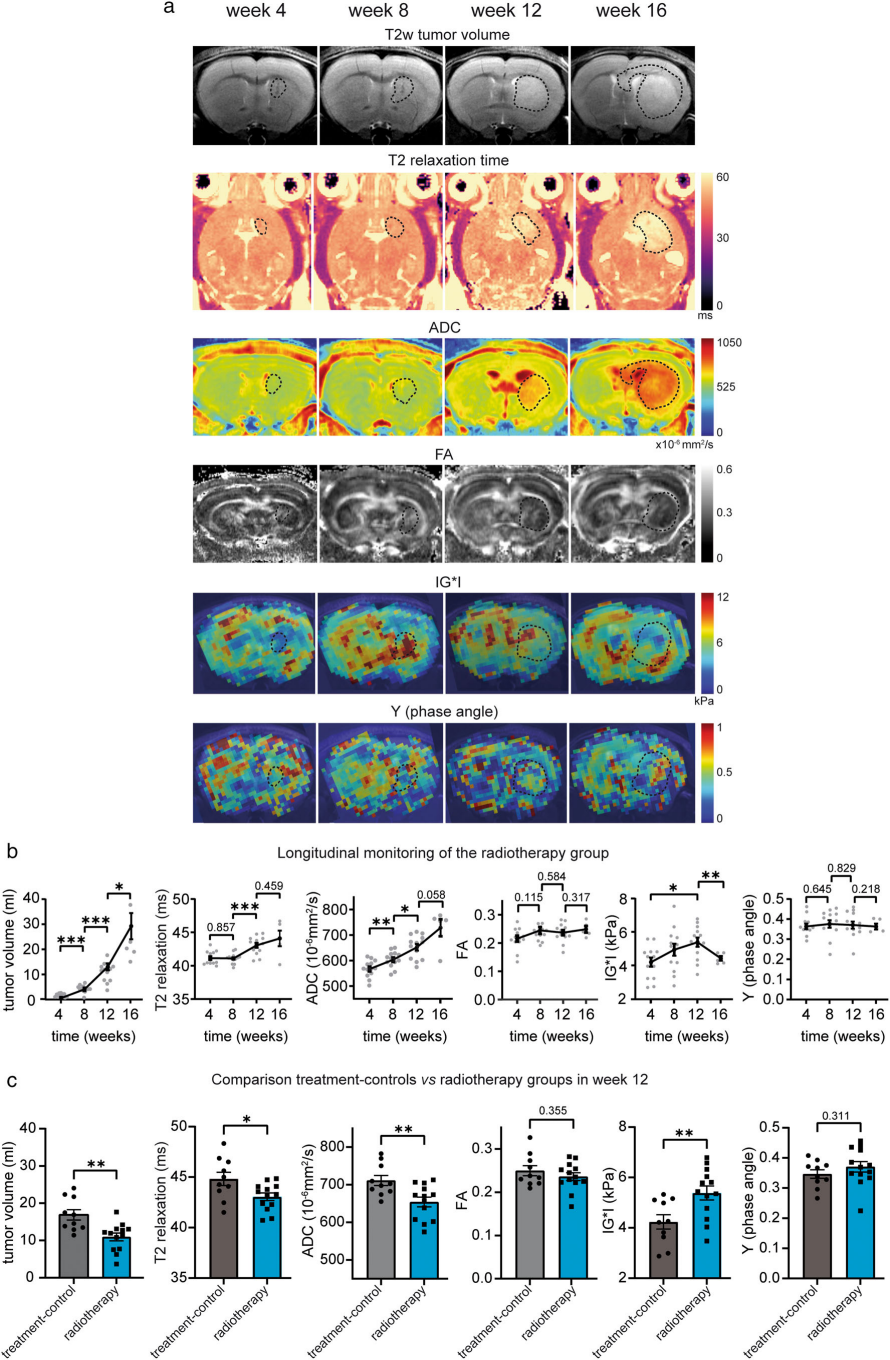
Analysis of T2 relaxation time, DTI‐ and MRE‐metrics in the tumor core of irradiated tumor‐bearing mice. (**a**) Representative T2w images, T2 relaxation maps, ADC and FA maps and elastograms of IG*I and Y of the radiotherapy group from week 4 to week 16, dotted lines indicate the tumor core. (**b**) Longitudinal quantification of T2w tumor volume, T2 relaxation time, ADC, FA, IG*I, and Y from week 4 to week 16. (**c**) Comparison of T2w tumor volume, T2 relaxation time, ADC, FA, IG*I, and Y between irradiated animals and treatment‐controls in week 12. Statistical analysis was performed by paired *t*‐tests when comparing different time points within a group and unpaired *t*‐tests when comparing different groups, **P ≤ 0.01; ***P ≤ 0.001, N = 13 mice (radiotherapy group), N = 10 mice (treatment‐controls).

Radiotherapy significantly decelerated tumor growth (tumor core volume after radiation in week 12: 10.96 ± 1.04 mm^3^ vs. 16.88 ± 1.40 mm^3^ in treatment‐controls; Fig. 6a–c). However, a long‐term stabilization or tumor regression was not achieved. Instead, a significant tumor growth was detected in the irradiated subgroup from week 12 to 16 with volumes matching those of treatment‐controls in week 12 (mean tumor core volume of irradiated mice in week 16: 23.01 ± 3.89 mm^3^ vs. 16.88 ± 1.40 mm^3^ in treatment‐controls in week 12, *P* = 0.088).

Similar to the tumor volume, a delayed increase in T2 relaxation time was observable in week 12 (T2 relaxation time in the radiotherapy group: 43.06 ± 0.37 msec vs. in treatment‐controls: 44.83 ± 0.66 msec) with a non‐significant continuation in week 16 (44.10 ± 1.17 msec; Fig. 6a–c). Moreover, the ADC increase was significantly decelerated after irradiation (ADC in week 12 in irradiated mice: 654.0 ± 9.22 × 10^−6^ mm^2^/s vs. 711.2 ± 13.42 × 10^−6^ mm^2^/s in treatment‐controls; Fig. 6a,c) and only continued slowly and non‐significantly (week 16: 730.5 ± 33.27 × 10^−6^ mm^2^/s, *P* = 0.058 when comparing weeks 12 and 16; Fig. 6b). The FA of the tumor core was not affected by radiotherapy as it remained stable throughout longitudinal monitoring (week 4: 0.215 ± 0.011, week 8: 0.244 ± 0.012, week 12: 0.236 ± 0.009, week 16: 0.248 ± 0.012; week 4 vs. 8: *P* = 0.115, week 8 vs. 12: *P* = 0.0.584, week 12 vs. 16: *P* = 0.317; Fig. 6a,b).

Tumor core stiffness progressively increased and was significantly higher after irradiation compared to week 4 (IG*I in week 4: 4.2 ± 0.27 kPa, week 8: 4.94 ± 0.36 kPa, week 12: 5.38 ± 0.28 kPa; Fig. 6a,b) and also compared to treatment‐controls (week 12: 5.38 ± 0.28 kPa vs. treatment‐controls: 4.24 ± 0.29 kPa; Fig. 6c). This was followed by a delayed, significant stiffness drop in week 16 (4.42 ± 0.14 kPa; Fig. 6b). The phase angle remained stable over time and was comparable to treatment‐controls (Fig. 6a–c).

When comparing the callosal imaging parameters in week 12 between the radiotherapy and treatment‐control group, no significant differences were detected (in irradiated animals: T2 relaxation time 40.69 ± 0.22 msec, ADC 608.7 ± 10.57 × 10^−6^ mm^2^/s, FA 0.404 ± 0.005, |G*| 5.65 ± 0.39 kPa, Y 0.344 ± 0.013 vs. in treatment‐controls: T2 relaxation time 41.13 ± 0.27 msec, ADC 633.4 ± 11.91 × 10^−6^ mm^2^/s, FA 0.39 ± 0.014, |G*| 4.73 ± 0.37 kPa, Y 0.321 ± 0.013; *P* = 0.213 for T2 relaxation time, *P* = 0.137 for ADC, *P* = 0.291 for FA, *P* = 0.11 for IG*I and *P* = 0.242 for Y).

**FIGURE 7:**
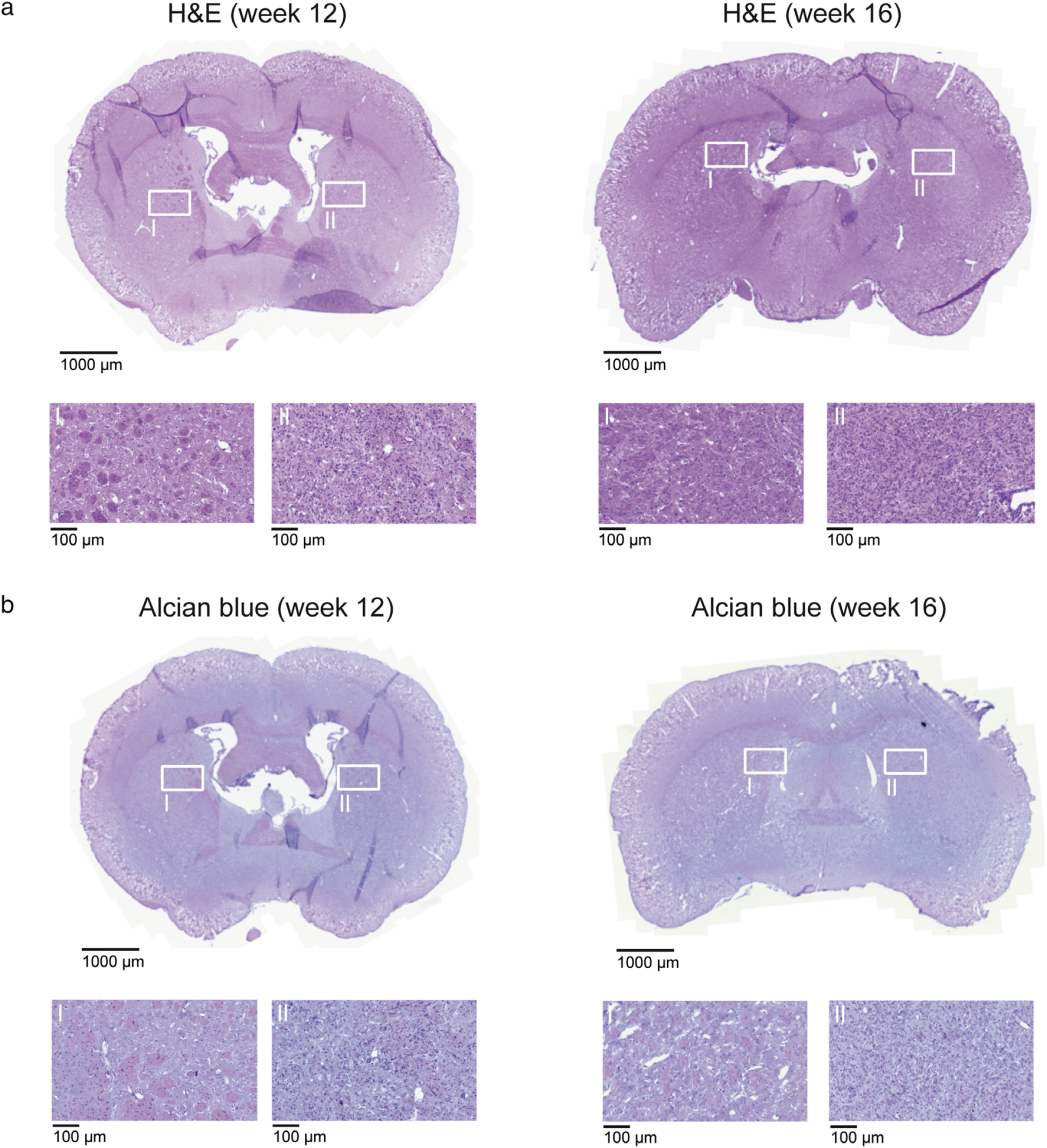
Histology of tumor‐bearing irradiated mice from week 12 and week 16. (**a**) H&E staining of irradiated mice from week 12 and week 16 with magnified illustrations of the tumor core center and the contralateral hemisphere. (**b**) Alcian blue staining of irradiated mice from week 12 and week 16 with magnified illustrations of the tumor core center and the contralateral hemisphere.

When correlating these findings to histology and comparing them to untreated animals in week 12, a lower tumor cell density (average cell density in the tumor core on a representative slice = 53.0 cells/100 μm^2^ vs. 59.9 cells/100 μm^2^) in treatment‐controls in week 12 and a more preserved ECM within the tumor core (percentage of disrupted ECM = 8.36% vs. 11.27% in treatment‐controls) was observed. Histologic findings in week 16 on the other hand outmatched those of untreated animals in week 12 (average cell density in the tumor core on a representative slice = 74.3 cells/100 μm^2^, percentage of disrupted ECM = 6.71%; Fig. 7a,b).

## Discussion

MRI sequences in clinical practice have shortcomings with regard to the detection and delineation of tumor cell invasion.^3^ MRE is a promising tool to supplement this diagnostic dilemma as microstructural tissue changes associated with cancer progression and invasion affect the biomechanical properties.^17^

In the current study, we demonstrated that early callosal stiffening correlated with tumor cell invasion, while structural and quantitative MRI did not show evident alterations at this time point. During invasion of S24 glioma cells, the structural integrity of the brain tissue is preserved as tumor cells invade along fiber tracts or blood vessels.^18^ Moreover, S24 glioma has been shown to progressively develop TM networks during the migration and invasion process.^6^ The presence of connected S24 glioma cells within the structurally intact callosal tissue is presumably the cause of the observed callosal stiffening. Results from 2D shear rheometry and 3D multifrequency MRE experiments revealed an association between increasing network density and an increasing elasticity parameter^34^ supporting this hypothesis. This theory is further supported by findings in the G9 PDX glioma model.^25^ Anti‐angiogenic treatment of G9 glioma led to a normalization of the tumor vasculature and a better preservation of normal brain architecture within the tumor core compared to untreated controls. This in turn was assumed to be the reason for treated tumors being stiffer than untreated tumors.^25^ FA did not capture tumor cell invasion, likely because callosal anisotropy was preserved due to the orientation of S24 cells along fiber tracts.

Our theory can also explain the evolution of tumor core stiffness: The increase in |G*| from week 4 to week 8 can be ascribed to the presence of S24 cells with abundant interconnecting TMs that respect the underlying structural organization of the brain as was perceptible on H&E and Alcian blue staining. The following tumor core softening is then caused by a destruction of the normal parenchymal organization with higher density of unconnected S24 cells, disrupted ECM and destroyed fiber tracts. In week 12, cores of S24 glioma were significantly softer than healthy brain tissue. This is in line with previous studies investigating the biomechanical properties of different experimental glioma, including U87MG, G9 and G30 PDX glioblastoma, RG2 rat glioma, D‐212‐MG PDX pediatric giant‐cell glioblastoma and DBT glioma.^25^, ^35^, ^36^ In all of these studies, established tumors with a volume of at least 20 mm^3^ ^25^, ^35^, ^36^ or demonstrating clear growth on structural MRI^27^, ^37^ were softer than healthy brain tissue. In week 12, the volume of S24 glioma approximated the tumor volumes reported in these previous studies. The phase angle Y did not change over time, meaning that the elastic and viscous contributions to the shear modulus change in the same direction. This is also in agreement with findings from others, as both shear and loss modulus of U87MG, RG2, and D‐212‐MG glioma were lower than in healthy brain tissue.^35^, ^36^

Overall, mean tumor core biomechanics are influenced by many factors, including cellularity,^35^ hemorrhage and necrosis,^27^ ECM integrity,^36^ inflammation or cellular perturbation in the tumor microenvironment^38^ and edema. These factors also led to a significant increase in T2 relaxation time (water content/edema) and ADC (cellularity and water content/edema) but cannot be completely disentangled based on their MRI‐and MRE‐signature. Regarding our findings for ADC, it is important to note that our DTI sequence was acquired at an experimental scanner with very high gradient strength and a relatively short diffusion time and thus predominantly reflects intracellular diffusion processes, whereas diffusion imaging at clinical scanners mainly reflects extracellular diffusion.^39^ While the increased cell density in glioblastomas imaged in clinical routine leads to an ADC decrease, the observed ADC increase in our model may thus be explained by the relatively large S24 cells (cell radii ~10 μm)^5^ in addition to an increased water content within the tumor core as was reflected by an increased T2 relaxation time.

Radiotherapy decelerated but did not halt tumor progression. This was reflected in a slower increase in tumor volume, tumor core T2 relaxation time and ADC as well as a delayed tumor core softening when comparing the temporal evolution of these parameters in treatment‐controls and in irradiated tumors. Histologically, a lower cell density and a more preserved ECM were observed in irradiated tumors in week 12 compared to treatment controls, also supporting our hypothesis, that the degree of tumor‐induced brain architectural disruption is reflected by |G*|. Even more so as the lower cell density is likely due to the ablation of unconnected tumor cells by radiotherapy while connected tumor cells are therapy‐resistant.^5^ Even though the differences in callosal T2 relaxation time, ADC and IG*I between irradiated mice and treatment‐controls in week 12 were non‐significant, the tendencies mirrored the effects observed in the tumor core. This can be explained by the experimental set‐up where most of the CC was not included in the irradiation‐field and thus not affected by any treatment‐effects.

In our sham‐control group, no alterations of DTI‐ and MRE‐derived parameters were observed, neither at the needle tract, nor within the corpus callosum. This indicates that ADC, FA, and tissue biomechanics are not altered by stereotactic injections *per se* but that our findings in tumor‐bearing animals are indeed due to S24 tumor growth.

### Limitations

First, due to our multiparametric imaging protocol, MRE acquisition was limited to nine slices and did not cover the entire brain. Consequently, at later time points large tumors and their infiltrative zones were only partially assessable by MRE. Second, MRI/MRE‐ and LSM‐data were manually or fiducial‐based co‐registered, and the tumor core manually segmented. There is thus the possibility of an examiner‐dependent bias which however would have affected all subgroups equally. Third, cellular resolution and thus the detection of individual, invading tumor cells is not achievable by the spatial resolution of MRE. Nonetheless, as demonstrated in our study, the biomechanical effect on the brain parenchyma exceeds the cellular level and is therefore detectable by MRE. Lastly, our results should be validated in other glioma models as well as by clinical investigations.

## Conclusion

In this study, MRE was sensitive to LSM‐proven early tumor cell infiltration, which was occult to structural and quantitative MRI. Imaging characteristics of the tumor core could be related to histopathological findings. Hence, tissue biomechanics render valuable information on glioma during progressive growth and under therapeutic pressure that are complimentary to clinically used MRI sequences. Therefore, MRE is a promising tool in the neurooncological setting and may allow a more precise delineation of the invasion zone and could further advance therapy monitoring.

## Supporting information


**Figure S1:** DTI‐ and MRE‐metrics in PBS sham‐injected mice. (a) Exemplary segmentation of the needle tract, the contralateral side, and the CC. (b) Longitudinal T2w images. (c) ADC and FA maps and elastograms of IG*I and Y, the injection site, an equally sized region in the contralateral hemisphere and the CC are indicated by the dotted lines. Visually, the needle tract does not lead to any alterations. (d) Longitudinal quantification of ADC, FA, IG*I and Y of the injection site. (e) Comparison of ADC, FA, IG*I and Y between the injection site and the contralateral region in week 4. Statistical analysis was performed using paired *t*‐tests. N = 9 mice for comparisons in week 4 and N = 4 for longitudinal comparisons (non‐irradiated sham‐injected animals).

**Data S1:** Supporting Information.
