# Supplementary figures and images for: Assessment of Tumor Cell Invasion and Radiotherapy Response in Experimental Glioma by Magnetic Resonance Elastography
Source: J Magn Reson Imaging. 2024 Aug 23;61(3):1203–18. doi: 10.1002/jmri.29567 (PMC11803692; doi:10.1002/jmri.29567)

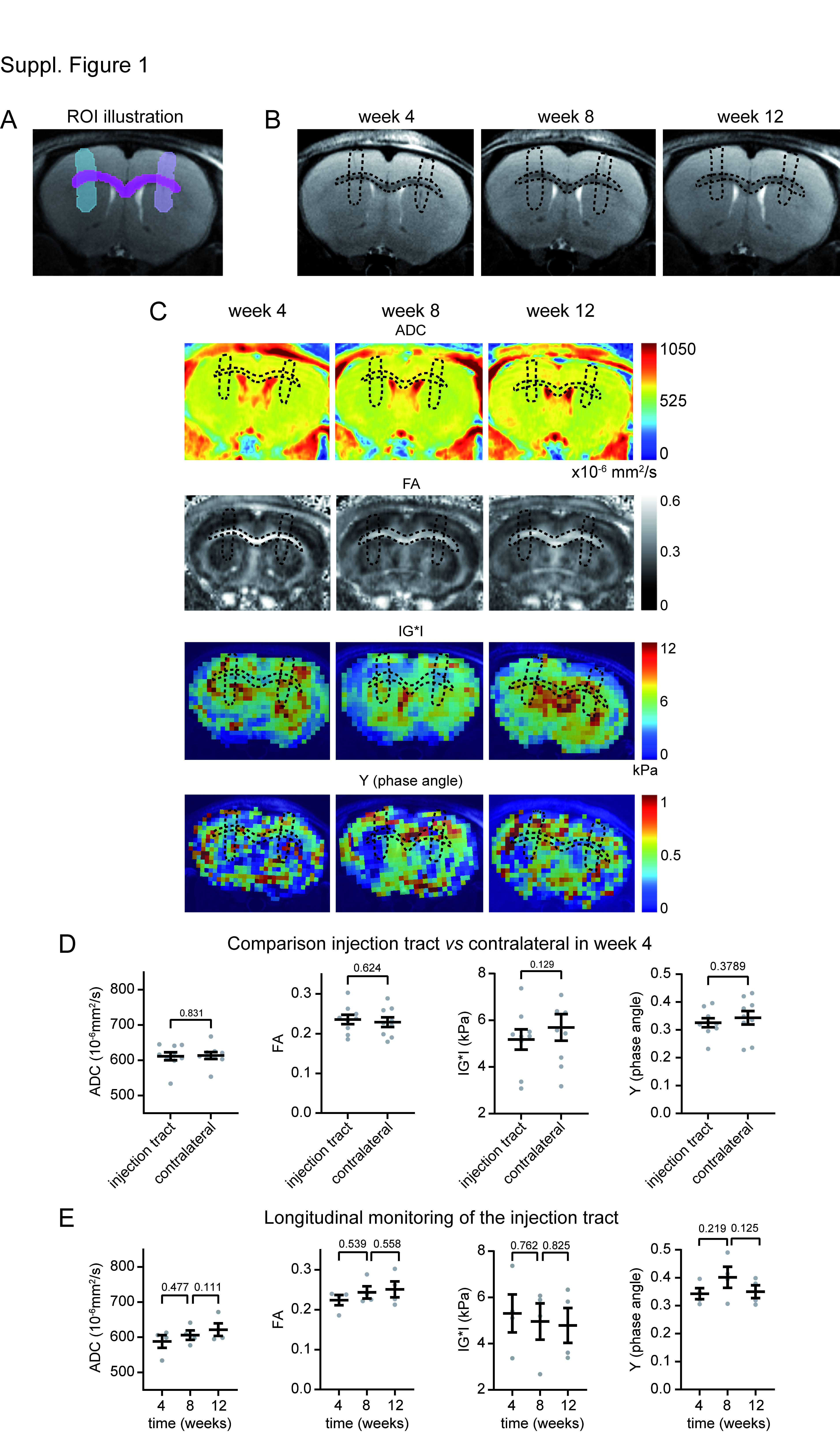

Supplement: Supplementary file 1 — Figure S1: DTI‐ and MRE‐metrics in PBS sham‐injected mice. (a) Exemplary segmentation of the needle tract, the contralateral side, and the CC. (b) Longitudinal T2w images. (c) ADC and FA maps and elastograms of IG*I and Y, the injection site, an equally sized region in the contralateral hemisphere and the CC are indicated by the dotted lines. Visually, the needle tract does not lead to any alterations. (d) Longitudinal quantification of ADC, FA, IG*I and Y of the injection site. (e) Comparison of ADC, FA, IG*I and Y between the injection site and the contralateral region in week 4. Statistical analysis was performed using paired t‐tests. N = 9 mice for comparisons in week 4 and N = 4 for longitudinal comparisons (non‐irradiated sham‐injected animals). [file JMRI-61-1203-s002.tif]
