# Supplementary material for: Assessment of Tumor Cell Invasion and Radiotherapy Response in Experimental Glioma by Magnetic Resonance Elastography
Source: J Magn Reson Imaging. 2024 Aug 23;61(3):1203–18. doi: 10.1002/jmri.29567 (PMC11803692; doi:10.1002/jmri.29567)
Supplement: Supplementary file 2 — Data S1: Supporting Information. [file JMRI-61-1203-s001.docx]

**Assessment of tumor cell invasion and radiotherapy response in experimental glioma by Magnetic Resonance Elastography**

**Supplementary Information**

***Radiotherapy***

The radiotherapy group received 6 Gy tumor irradiation on 3 consecutive days (cumulative dose of 18 Gy). For this, mice were anaesthetized with ketamine and xylazine and fixed in a custom-made radiation apparatus to insure persistence in the correct position for irradiation of the tumor region only. The surrounding brain parenchyma and the body were shielded mimicking stereotactic radiotherapy in patients. Irradiation was performed using a MultiRad Faxitron 225 as previously described (1). In line with previous work (1, 2), irradiation took place in week 9 after tumor cell implantation as tumor establishment has been demonstrated for this time point. Yet unpublished own data showed that the administrated irradiation scheme of 3 x 6 Gy is effective in reducing the tumor cell count without a complete eradication of the tumor, mimicking the human disease course while the radiotherapy related toxicity is low. Nonetheless, treated mice underwent close clinical monitoring and weight monitoring and received supportive therapy such as subcutaneous sodium chloride injections and/or high fat diet if needed.

***Rehydration And Histology***

The rehydration process started with reversed incubation in 100%, 80%, 60% and 40 % methanol (catalog # 4627; Carl Roth) and in PBS with gentle shaking for 1h. In a second step, overnight fixation of the samples at 4°C in 4% PFA was performed. The next day, samples were washed twice with PBS for 30 min each, then placed in 30% sucrose solution for 24 to 48 hours, embedded in TissueTek (REF#4583, Sakura) and frozen on dry ice. Cryo-sectioning of the tissue samples was performed at a slice thickness of 10 µm. For hematoxylin & eosin (H&E) staining, slides were washed with distilled water, stained with hematoxylin (catalog #9194, Carl Roth) for 6 min, rinsed with tap and distilled water and then eosin-counterstained for 3 min (catalog #9194, Carl Roth). Slides were successively immersed in 70%, 96% and 100% ethanol for a duration of 2 min each followed by xylene and then mounted.

The Alcian blue staining was performed with a staining kit and following the manufacturer protocol (catalog #1326570001, Sigma-Aldrich). After rinsing with distilled water, slides were stained with Alcian blue solution (pH 2.5) for 15 min and then again rinsed, first in running tap, then in distilled water. Slides were placed in 0.1 % nuclear fast red-aluminum sulfate solution for 10 min followed by immersion in an ascending alcohol series of 70%, 96% and 100% ethanol (2 min at each concentration). This was followed by xylene immersion and the mounting of the slides.

For immunofluorescence (IF) staining, the GFP signal was enhanced by counterstaining against GFP while slices were free floating on a shaker at 45r/min. Blocking was conducted with 10% Bovine Serum/0.3% tween for 1h at room temperature. Subsequently, an anti-GFP antibody (chicken anti-GFP polyclonal, 1:200, Abcam ab13970) was added and left to incubate overnight at 4°C. After 3x PBS wash for 5 min each, the secondary antibody (Alexa488 goat anti-chicken, 1:500, A11039 Invitrogen) was added and left to incubate for 1h at room temperature. After additional 3x PBS washes for 5 min each, slices were covered/mounted with Vectashield/DAPI mounting medium and stored at 4°C until imaging.

**References:**

1. Osswald M, Jung E, Sahm F, et al.: Brain tumour cells interconnect to a functional and resistant network. *Nature* 2015; 528:93–98.

2. Jung E, Osswald M, Ratliff M, et al.: Tumor cell plasticity, heterogeneity, and resistance in crucial microenvironmental niches in glioma. *Nat Commun* 2021; 12:1014.
